# The epidemiological and genetic characteristics of human parvovirus B19 in patients with febrile rash illnesses in China

**DOI:** 10.1038/s41598-023-43158-y

**Published:** 2023-09-23

**Authors:** Haoran Jiang, Qi Qiu, Yangzi Zhou, Yan Zhang, Wenbo Xu, Aili Cui, Xiaomei Li

**Affiliations:** 1 National Health Commission (NHC) Key Laboratory of Medical Virology and Viral Diseases, WHO WPRO Regional Reference Measles/Rubella Laboratory, National Institute for Viral Disease Control and Prevention, Chinese Center for Disease Control and Prevention, Beijing, 102206 People’s Republic of China; 2 School of Public Health and Management, Shandong First Medical University & Shandong Academy of Medical Sciences, Jinan, 250117 People’s Republic of China; 3 Shanghai Municipal Center for Disease Control and Prevention, Institute of Infectious Disease Prevention and Control, 1380 Zhongshan West Road, Xuhui District, Shanghai, 200336 People’s Republic of China; 4 NHC Key Laboratory of Systems Biology of Pathogens, Institute of Pathogen Biology, Chinese Academy of Medical Sciences & Peking Union Medical College, Beijing, 100730 People’s Republic of China

**Keywords:** Evolutionary biology, Genetic variation, Gene amplification, DNA sequencing, Epidemiology

## Abstract

To understand the epidemiological and genetic characteristics of B19V, a multiple-province surveillance of patients with febrile rash illnesses (FRIs) were conducted in China during 2009 ~ 2021. The clinical specimens of 3,820 FRI patients were collected and tested for B19V DNA. A total of 99 (2.59%) patients were positive for B19V, and 49 (49.49%) were children under 5 years old. B19V infections occurred throughout the year without obvious seasonal pattern. Ten NS1-VP1u sequences and seven genome sequences were obtained in this study, identified as subgenotype 1a. Combined with the globally representative genome sequences, no temporal and geographic clustering trends of B19V were observed, and there was no significant correlation between B19V sequences and clinical manifestations. The evolutionary rate of the B19V genome was 2.30 × 10^–4^ substitutions/site/year. The number of negative selection sites was higher than that of positive selection sites. It was the first to comprehensively describe the prevalence patterns and evolutionary characteristics of B19V in FRI patients in China. B19V played the role in FRI patients. Children under 5 years old were the main population of B19V infection. Subgenotype 1a was prevalent in FRI patients in China. B19V showed a high mutation rate, while negative selection acted on the genome.

## Introduction

As a member of the family *Parvoviridae*, human parvovirus B19 (B19V) was a global and common infectious pathogen in humans^1^. B19V was first identified by Cossart et al.^2^ during the evaluation of tests for hepatitis B virus surface antigen in 1975. Subsequently, B19V infection has been reported worldwide. The transmission of B19V infection usually occurs through the respiratory route, and infections can also be transmitted vertically from mother to fetus and through the transfusion of blood products and bone marrow transplants^3–5^. B19V infection is common in childhood, and it can also occur throughout adulthood albeit at a lower rate^6^. The findings showed a significant negative correlation between viremia rates and age, and the positive rate of B19V DNA decreased with age, from 2.24% in 19–30 years to 0.87% in 41–50 years^7,8^. However, the prevalence of IgG antibodies directed against B19V ranges from 2 to 15% in children 1 to 5 years old, 15 to 60% in children 6 to 19 years old, 30 to 60% in adults, and more than 85% in the geriatric population^9–12^. The manifestations of B19V infection depend on the age, immunity and hematologic status of the host^1,13^. Most B19V infections are asymptomatic. The common clinical manifestation of B19V infection is erythema infectiosum in children, while arthropathy is a more common manifestation of infection in adults, particularly in women^14^. In immunocompromised hosts, persistent B19V infection presents with pure red cell aplasia and chronic anemia^13,15^. B19V is now recognized as the only etiologic agent of erythema infectiosum^16–18^. Due to similar eruption symptoms, erythema infectiosum can be confused with rubella^19–22^.

B19V is a small nonenveloped single-stranded DNA virus with an approximately 5.5-kb long genome. This DNA encodes six viral proteins, among which the three major proteins are nonstructural protein 1 (NS1), viral protein 1 (VP1) and viral protein 2 (VP2). In addition, there are three small nonstructural proteins of 7.5 kDa, X, and 11 kDa. Based on the phylogenetic analysis of the NS1-VP1u region, B19V was classified into three genotypes: genotype 1, genotype 2, and genotype 3^23^. With the increasing number of genome sequences obtained, B19V was further subdivided into subgenotypes, which were useful for molecular epidemiological studies. Genotype 1 was further divided into two subgenotypes, 1a and 1b, and genotype 3 was divided into two subgenotypes, 3a and 3b^24,25^.

To date, investigations of B19V have been carried out by many researchers in recent years, including in China. However, most of them focused on viral infection in blood products from healthy donors, organ transplantation and the status of B19V infection in pregnant women^26–30^. There have been few studies on the prevalence of B19V in patients with febrile rash illnesses (FRIs). From January 2009 to June 2021, active surveillance of FRIs was conducted in ten provinces of China, including Anhui, Beijing, Hebei, Henan, Hunan, Shandong, Shanxi, Shaanxi, Shanghai and Xinjiang Uygur Autonomous Region. Multiple viral pathogens were tested in the clinical specimens obtained from FRI patients, including measles virus, rubella virus, human enterovirus, varicella-zoster virus, dengue virus, B19V, Epstein‒Barr virus, and human herpes virus 6, which covered the most common viral pathogens of FRIs. In this study, the epidemiological and genetic characteristics of B19V were comprehensively analyzed based on the surveillance data of FRI patients in ten provinces in China from 2009 to  2021.

## Results

### The prevalence of B19V in FRI patients

In total, the clinical specimens of 3,820 FRI patients were tested for B19V DNA during 2009 ~ 2021, of which 99 were positive for B19V (Table 1). The positive rate of B19V was 2.59% in FRI patients. The positive rate of B19V was significant higher in females than in males (*p* = 0.040). The median age of B19V-positive patients was 5 years old (IQR: 1–20). Among the 99 B19V-positive patients, 49 cases (49.49%) were children under 5 years old, 31 cases (31.31%) were adolescents aged 5–17 years old, 19 cases (19.19%) were adults aged 18–59 years old, and no B19V-positive patient was detected in the elderly aged ≥ 60 years old. The positive rate was highest in the age group of 5 ~ 17 years old (3.59%), followed by < 5 years old (2.57%), 18–59 years old (2.08%), and ≥ 60 years old (0.00%). There was a significant difference in the positive rates of B19V in different age groups (*p* = 0.046). B19V was detected in FRI patients throughout the year, with no obvious seasonal pattern (*p* = 0.155). The positive rate of B19V in autumn was highest (3.51%), followed by winter (3.23%), summer (2.70%) and spring (2.02%). The B19V-positive rates in FRI patients were slightly higher in autumn and winter than in spring and summer, but the difference was not significant. In terms of regional distribution, the positive rate of B19V in FRI patients in Northern China (3.50%) was higher than that in Southern China (0.44%), and the difference was statistically significant (*p* = 0.001).

**Table 1:** Epidemiological characteristics of B19V-positive patients with FRI.

Variables		Number of FRI patients	Number of B19V-positive patients	Positive rate (%)	χ2	*P value*
Total		3820	99	2.59		
Gender	Male	2160	46	2.13	4.203	0.040
	Female	1660	53	3.19		
Age group (years)	< 5	1906	49	2.57	8.006	0.046
	5 ~ 17	864	31	3.59		
	18 ~ 59	912	19	2.08		
	≥ 60	138	0	0.00		
Season*	Spring	1736	35	2.02	5.239	0.155
	Summer	963	26	2.70		
	Autumn	626	22	3.51		
	Winter	495	16	3.23		
Region	Northern	2684	94	3.50	29.646	0.001
	Southern	1136	5	0.44		

*Spring: March ~ May; Summer: June ~ August; Autumn: September ~ November; Winter: December ~ February.

### Genotype identification and phylogenetic analysis of B19V based on the NS1-VP1u region

A total of 10 NS1-VP1u sequences were obtained from the clinical specimens of 99 FRI patients with B19V infection in this study, including 4 from Shanxi, 3 from Shanghai, 2 from Shaanxi, and 1 from Henan. These sequences were preliminarily identified by a BLAST search in the NCBI Nucleotide GenBank database (https://blastncbi.nlm.nih.gov/) as subgenotype 1a of genotype 1, and the nucleotide similarity was 98.7% ~ 100.0%.

**Figure 1:**
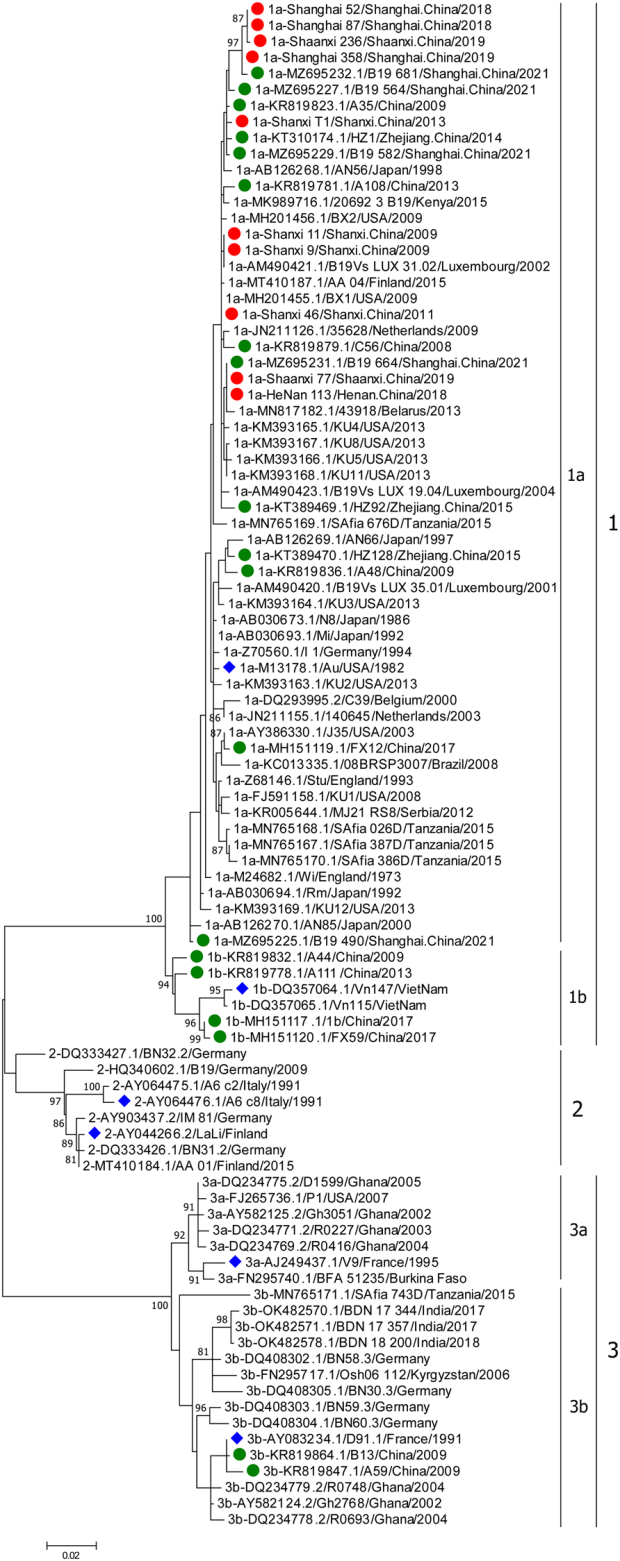
Phylogenetic tree of the B19V sequence based on the NS1-VP1u region. A phylogenetic tree was constructed with the maximum likelihood method. Bootstrap values above 80% are shown (1000 replicates). The blue diamond represents the reference sequences of genotype or subgenotype; the green solid circle represents the Chinese representative sequences; the red solid circle represents the sequence obtained in this study.

Based on the phylogenetic tree constructed with the NS1-VP1u dataset, B19V sequences could be divided into three genotypes (Fig. 1). The average inter-genotype P-distances of B19V ranged from 0.069 to 0.103, and the average intra-genotype P-distances were 0.015 for genotype 1, 0.021 for genotype 2, and 0.025 for genotype 3. The average inter-subgenotype P-distances in the same genotype were 0.031 ~ 0.032, and the average intra-subgenotype P-distances were 0.011 ~ 0.022. Combined with the globally representative B19V sequences downloaded from GenBank, no trend in temporal or geographical clustering of B19V was observed in the phylogenetic tree. In addition, the sequences obtained from healthy donor blood and the clinical specimens of the B19V patients with different clinical manifestations were also interleaved in the phylogenetic tree.

A total of 29 Chinese representative sequences of B19V were analyzed in this study, including 10 sequences from FRI patients and 19 sequences downloaded from GenBank. The 29 Chinese sequences belonged to subgenotypes 1a, 1b and 3b, of which 79.31% (23/29) were subgenotype 1a (Supplementary Table S1). All sequences obtained from FRI patients belonged to subgenotype 1a. In addition, the sequences of subgenotype 1a were also obtained from healthy donor blood and the clinical specimens of patients with kidney transplants, HIV, and pneumoniae, while the sequences of subgenotypes 1b and 3b were all sourced from healthy donor blood.

### Genetic characteristics of the B19V genome

A total of 7 B19V genome sequences were obtained in this study, including 2 from Shanxi, 2 from Shanghai, 2 from Shaanxi, and 1 from Henan. The 7 genome sequences all belonged to subgenotype 1a, and the nucleotide similarity was 98.5% ~ 99.7%. The phylogenetic tree was conducted based on the genome dataset of B19V. The topologies of these sequences were almost identical in the ML trees of the genome and NS1-VP1u region (Supplementary Figure S1). The average intergenotype P-distances ranged from 0.090 to 0.130, and the average intragenotype P-distances of B19V were 0.017 for genotype 1, 0.026 for genotype 2, and 0.041 for genotype 3. The average inter-subgenotype P-distances in the same genotype were 0.050 ~ 0.055, and the average intra-subgenotype P-distances were 0.016 ~ 0.031. In terms of genetic distance, genotype 1 was far from genotypes 2 and 3, while genotype 3 showed higher genetic diversity than genotype 1 and genotype 2. The genetic diversity of genotype 1 was the lowest, although it was widely prevalent in the world.

There were some genotype-specific amino acid sites in the six proteins of B19V, such as 26 in NS1, 7 in 7.5 kDa, 10 in VP1, 2 in X, 4 in VP2, and 13 in 11 kDa (Supplementary Table S2). The amino acid mutation rates of the six proteins from high to low were 11 kDa (39.36%, 37/94), X (23.46%, 19/81), 7.5 kDa (18.92%, 14/74), NS1 (12.67%, 85/671), VP1 (11.78%, 92/781) and VP2 (8.30%, 46/554). The amino acid mutation rates of the three major proteins were lower than those of the three small proteins.

### Evolutionary rate and selection analysis of the B19V genome

Because 6 sequences missed the date information of case onset and sample collection, 52 out of 58 sequences in the genome dataset were used for analysis of B19V. By using BEAST package (version 1.10.4), no time trend for B19V was observed in the BEAST tree (Supplementary Figure S2). And, the evolutionary rate of the B19V genome was estimated to be 2.30 × 10^–4^ substitutions/site/year (95% HPD: 1.60 × 10^–4^ ~ 3.04 × 10^–4^ substitutions/site/year). Among them, the evolutionary rate of the subgenotype 1a B19V genome was 2.64 × 10^–4^ substitutions/site/year (95% HPD: 2.05 × 10^–4^ ~ 3.26 × 10^–4^ substitutions/site/year). In the B19V genome, the evolutionary rates and nucleotide similarity of genes encoding different proteins were variable. The evolutionary rates of NS1, VP1 and VP2 were similar, slightly higher than that of the B19V genome. 11 kDa was the fastest evolving small nonstructural protein in the genome, with an evolutionary rate of up to 3.58 × 10^–4^ substitutions/site/year. The 7.5 kDa protein was the slowest evolving protein in the genome, with an evolutionary rate of 9.03 × 10^–5^ substitutions/site/year (Table 2).

**Table 2:** Evolutionary analysis based on the different coding regions of B19V.

Gene	Nucleotide substitution model ^a^	Clock model ^b^	Evolutionary rates (95%HPD)
NS1	GTR + G	Uncorrelated lognormal relaxed clock	2.50 × 10^–4^ (1.47 × 10^–4^ ~ 3.61 × 10^–4^)
7.5 kDa	HKY + I	Uncorrelated exponential relaxed clock	9.03 × 10^–5^ (8.35 × 10^–6^ ~ 1.96 × 10^–4^)
VP1	GTR + G	Uncorrelated lognormal relaxed clock	2.57 × 10^–4^ (1.77 × 10^–4^ ~ 3.45 × 10^–4^)
X	K80 + I	Strict clock	2.72 × 10^–4^ (9.99 × 10^–5^ ~ 4.59 × 10^–4^)
VP2	TrN + I + G	Strict clock	2.61 × 10^–4^ (1.92 × 10^–4^ ~ 3.29 × 10^–4^)
11 kDa	HKY + G	Strict clock	3.58 × 10^–4^ (1.61 × 10^–4^ ~ 5.70 × 10^–4^)

a: the best model for nucleotides was predicted by jModelTest2 on XSEDE v2.1.6; b: the best fit clock model was determined by stepping-stone sampling (SS).

To better understand the evolutionary dynamics of B19V, selection pressures on six proteins in the genome were also analyzed in this study. The ω values for six proteins were all less than 1, indicating that these proteins were all under negative selection pressure. Through the analysis with the FEL, SLAC and FUBAR methods, negative selection sites were found in the coding regions of six proteins, and their number was far higher than that of positive selection sites (Table 3). The number of negative selection sites in the coding regions of the three major proteins varied with different methods. In addition, through the analysis with the FEL, FUBAR and MEME methods, a small number of positive selection sites were also found in the coding genes of NS1, VP1 and VP2, with a P value of < 0.05 (MEME and FEL methods) or a posterior probability of > 0.95 (FUBAR method) (Table 3). A total of 4 positive selection sites were found in the NS1 protein, among which 3 sites (V151 → G, I181 → M/L, A206 → N) were located at the N-terminus of the NS1 protein (MEME), and one site (F554 → S) was located at the C-terminus of the NS1 protein (FUBAR). Notably, a nucleotide substitution (T1661C) in the NS1 protein (F554 → S) was found to be a nonsynonymous mutation with positive selection pressure, while the same substitution was a synonymous mutation at the first nucleotide of codon 65 (TTG → CTG) in the 7.5 kDa protein, where it was found to be subject to negative selection pressure. Four positive selection sites were also found in the VP1 protein, of which two sites (E4 → T/K/Q/N/A, P96 → S) were located in VP1u, and two sites (VP1:S548, T716 → S; VP2:S321, T489 → S) were located in the C-terminus of the VP1/VP2 protein (MEME). In particular, a highly active mutation was found at codon 4 in VP1u (FUBAR).

**Table 3:** Analysis of the selection pressure of B19V in the coding regions of six proteins.

Coding region	ENC ^a^	dS	dN	ω	Number of negatively selected codons ^b^			Number of positively selected codons ^c^			
					FEL	SLAC	FUBAR	FEL	SLAC	FUBAR	MEME
NS1	671	0.25963	0.01694	0.06525	235	75	297	0	0	1	3
7.5 kDa	74	0.03700	0.01744	0.47135	5	1	5	0	0	0	0
VP1	781	0.25393	0.01125	0.04430	268	136	355	1	0	1	2
X	81	0.05413	0.01931	0.35673	7	4	6	0	0	0	0
VP2	554	0.29603	0.00686	0.02317	242	125	309	0	0	0	2
11 kDa	94	0.21330	0.05830	0.27332	19	9	17	0	0	0	0

a: effective number of codons referring to the B19V sequence (accession No. NC_000883.2); b c: Number of negatively and positively selected codons obtained using the FEL, SLAC, FUBAR, and/or MEME methods implemented in DataMonkey.

## Discussion

B19V was one of the viral pathogens in FRI patients, especially for children under 5 years of age. It was frequently detected in measles- and rubella-negative patients in countries with measles/rubella elimination or near elimination^20^. For example, in Campinas, Brazil, a setting of low measles and rubella virus transmission, 2.4% of FRI patients under 40 years of age were laboratory confirmed to be positive for B19V^31^. In Bulgaria, 56.18% of measles- and rubella-negative patients were positive for B19V viral DNA, and 48.97% were positive for B19V-IgM^32^. In a survey of children under 15 years of age with measles- and rubella-like illness in Iran, 10.81% of children were positive for B19V viral DNA, while 19.21% and 18.87% were positive for B19V-IgM and B19V-IgG, respectively^20^. The positive rate of B19V among FRI patients varies widely and depends on geographical region, detection method, measles and rubella control status and age groups tested. In China, the positive rates of B19V in FRI patients ranged from 0.43 to 9.52%^33–37^. In this study, the positive rate of B19V in FRI patients was 2.59%, similar to the previous reports. Surveillance data in this study showed that nearly half of B19V patients were children under 5 years old. B19V infection in FRI patients occurred throughout the year without obvious seasonal patterns, but the B19V-positive rates in FRI patients were slightly higher in autumn and winter than in spring and summer in China. In contrast, a study in Iran found slightly different results from ours, as it found that the majority of B19V infections were concentrated between March and June, with May being the most dominant month of the year^20^. The positive rate of B19V in FRI patients in Northern China was significantly higher than that in Southern China. Since there are very few epidemiological studies on B19V in FRI patients in China and even in the world, further studies are needed to fully characterize the prevalence of B19V in the future.

To date, three genotypes of B19V have been recognized as distinct circulating lineages worldwide. Compared with genotypes 2 and 3, genotype 1 was the predominant genotype of B19V, circulating in all continents, especially subgenotype 1a. Similar to the predominant genotype of B19V circulating globally, most of the B19V sequences detected in China were genotype 1, including the sequences obtained in FRI patients in this study. So far, the Chinese B19V sequences of genotype 1 have been obtained from healthy donor blood and patients with FRIs, kidney transplants, HIV, etc. In addition, a few B19V sequences of subgenotypes 1b and 3b were detected from healthy donor blood^27^.

No B19V sequence of genotype 2 has been detected in China. Genotype 2 was sporadically detected in a few countries of Europe, including Finland, Germany, and Italy^38–40^. Because it was very rarely detected as circulating virus and frequently detected as persistent in tissues of elderly individuals, genotype 2 was considered an ancient genotype^41^. The data suggested that a global replacement of genotype 2 with genotype 1 may have occurred in the 1960s^41^. Genotype 3 was highly dominant in West Africa, which has been regarded as an endemic region for genotype 3 B19V infection^25^. However, it now tends to spread outside of Africa, especially subgenotype 3b, which is increasingly being found in some countries of Asia, Europe, and America^41^.

Despite being a DNA virus, B19V showed a high mutation rate. At the nucleotide level of the genome, the intergenotype genetic distances among the three genotypes of B19V ranged from 0.090 to 0.130. The phylogenetic analysis of B19V showed that genotype 1 was far from genotypes 2 and 3 in terms of genetic distance. Compared with genotypes 2 and 3, genotype 1 showed low diversity although a large number of B19V genotype 1 strains are widespread around the world. The genetic variation within genotype 1 is usually less than 2%, and that of genotype 2 and genotype 3 is usually between 3 and 10%, similar to the previous report^41^. Moreover, no temporal or geographic clustering trend among three genotypes was observed in phylogenetic tree of B19V.

B19V infection is associated with a variety of clinical manifestations. In addition to erythema infectiosum, B19V can also cause aplastic anemia, transient aplastic crisis, hepatic inflammation, myocarditis, arthritis, and neurological disorders. The clinical manifestations of B19V infection depend on the interplay among the pathogenetic potential of the virus, its adaptation to different cellular environments, and the physiological and immune status of the infected individuals^41,42^. The current evidence suggests that all genotypes have been found in patients with B19V-related symptoms as well as asymptomatic individuals^43^. In this study, all the B19V sequences obtained from the patients with FRIs belonged to genotype 1. Except for erythema infectiosum, genotype 1 could also be detected in patients with different B19V clinical manifestations, while genotype 3 was mostly detected in patients who had undergone liver/kidney transplantation or patients with leukemia^44,45^. Genotype 2 has only been detected in the bone marrow of older patients with cytopenias of unknown origin or chronic lymphocytic leukemia^44,46^. After acute infection, residual viral DNA of B19V can remain in tissue for decades or even throughout life^47,48^. However, no clear correlations were observed between sequences and clinical manifestations in the phylogenetic tree of B19V.

In the evolutionary analysis, the average evolutionary rate of the B19V genome in this study (2.30 × 10^–4^ substitutions/site/year) was similar to that of the subgenotype 1a B19V genome (2.64 × 10^–4^ substitutions/site/year). These two evolutionary rates were in the range of 10^–4^ substitutions/site/year, which was consistent with the previous studies^25,49–51^. The evolutionary rates of NS1, VP1 and VP2 were similar, slightly higher than that of the B19V genome. The two small nonstructural proteins, 11 kDa and 7.5 kDa, were the fastest and slowest evolving proteins in the genome, respectively. The amino acid mutation rates of the three major proteins were lower than those of the three small proteins, suggesting that the high mutation rate may enable progressive diversification of viral isolates^50,51^. The selection pressure analysis in this study revealed that ω values in different coding regions were less than 1. Negative selection acted on the B19V genome, including the six proteins, indicating that the evolution of B19V was under strong negative selection, similar to previously published results^49,50^. Only a few positive selection sites were found in NS1, VP1 and VP2 in this study. While no positive selection site was found in the functional domain of NS1 protein, including the RI motif-replication-initiator motif (codons 79–147), NTP-binding and helicase domains (codons 320–416), and putative transactivation domains: TAD1 (codons 416–424), TAD2 (codons 523–531), and TAD3 (codons 566–576)^50^. Four positive selection sites in VP1 codons (4, 96, 548 and 716) were found in the dominant VP1/VP2 immune epitopes (VP1-F1: aa 2–100, VP1-F3 = VP2: aa 227–781), which could elicit a long-lasting immune response^52^. These positive selection sites in the antigenic domain of VP1/VP2 may be driven by immune response pressures.

## Conclusions

B19V played the role in FRI patients in China. Children under 5 years old are the main population of B19V infection. Genotype 1 was prevalent in FRI patients with B19V infection in China, especially subgenotype 1a. B19V showed a high mutation rate in viral evolution, while negative selection acted on most of the B19V genome. These findings would provide a better understanding of the burden of B19V infections and the development of potential vaccines against B19V.

Through our multiple-province surveillance of FRI patients for more than 10 years, the data obtained in this study was the first to at least partially reflect the prevalence of B19V in FRI patients in China. However, due to the low viral yield in clinical samples and failure to grow in cell cultures of B19V, only a few NS1-VP1u sequences and genome sequences were obtained in FRI patients. The limited epidemiological data and sequences of B19V may not comprehensively reflect the prevalence of B19V in China. Therefore, a systematic and continuous surveillance of B19V is crucial in future.

## Materials and methods

### Ethics statement and case sources

Active FRI surveillance was conducted from January 2009 to June 2021 in multiple provinces in China. The surveillance enrolled FRI patients of all ages. The patients with the clinical manifestation of fever (> 37.5 °C, lasting more than one day) and systemic or localized skin or mucosal rash were enrolled in sentinel hospitals after they or their parents/guardians provided informed consent. Detailed clinical and epidemiological information was collected through a standardized case reporting form by the staff of sentinel hospitals. This study was approved by the Ethics Committee of the National Institute for Viral Disease Control and Prevention, Chinese Centers for Disease Control and Prevention and all methods were performed in accordance with the relevant guidelines. In this study, only clinical specimens including throat swabs or sera were collected and detected from FRI patients and no human experimentation was involved.

### The positive rate of B19V and statistical analysis

The epidemiological dataset of FRI patients with B19V infection was established and analyzed in this study. Because age data did not conform to a normal distribution, the age of FRI patients with B19V infection is presented as the median and interquartile range (IQR). The age groups were divided as follows: < 5, 5 ~ 17, 18 ~ 59, and ≥ 60 years old. In order to analyze the regional differences in the prevalence of B19V, ten provinces were divided into Northern China (Xinjiang Uygur Autonomous Region, Beijing, Hebei, Henan, Shaanxi, Shandong, Shanxi) and Southern China (Anhui, Hunan, Shanghai) (Supplementary Table S3). Descriptive statistical analyses were performed to analyze the positive rates of B19V by gender, age group, season and region during 2009 ~ 2021. Chi-square tests were used to analyze the significant differences in frequency data in SPSS 26.0, and *p* < 0.05 was considered to be statistically significant.

### Viral DNA extraction and PCR amplification

Clinical specimens, including throat swab or serum specimens, were collected from FRI patients. According to the manufacturer's instructions, viral DNA was extracted from clinical specimens of FRI patients by using a viral DNA/RNA nucleic acid extraction and purification kit (Xi'an Tianlong Technology Co., Ltd, China) and stored at −80 °C until testing. The preliminary screening of the eight FRI-associated viruses mentioned above was performed by real-time PCR^53^. The 994 nt NS1-VP1u region (positions 2117 to 3110 in NC_000883.2) and the near complete coding B19V genome (positions 616 to 5174 in NC_000883.2) were amplified by nested PCR from the B19V-positive specimens by using Platinum PCR SuperMix (Invitrogen, USA). The pairs of primers for NS1-VP1u and the genome are listed in Supplementary Table S4. The first and second rounds of PCR amplification were performed under the following conditions: denaturation at 94 °C for 4 min; amplification for 40 cycles of 94 °C for 1 min, 45 °C for 1 min, and 72 °C for 1 min 40 s; and extension for 10 min at 72 °C at the end of the reaction. The PCR-positive products of B19V were identified by 1.5% agarose gel electrophoresis and sequenced by Sangon Bioengineering (Shanghai) Co., Ltd. The raw B19V sequence was edited and assembled by Sequencher software (Version 5.4.5).

### Sequence datasets

A total of 85 representative NS1-VP1u sequences were downloaded from GenBank, which consisted of 6 reference sequences of the B19V genotype/subgenotype, 19 sequences from China, and 60 sequences from other countries. Fifty-one representative B19V genome sequences were downloaded from GenBank from 13 countries worldwide during 1973 ~ 2021. These representative sequences were derived from plasma from healthy blood donors and the clinical specimens of B19V patients with different clinical manifestations, including erythema infectiosum, arthritis, aplastic crisis, hepatic inflammation, myocarditis, etc.

Multiple nucleotide sequences, including the sequences obtained in this study and the representative sequences downloaded from GenBank, were aligned and edited using MAFFT software (Version 7.4.5) and MEGA software (Version 7.0). Subsequently, the NS1-VP1u dataset and the genome dataset were generated and used for genotype identification and viral evolutionary analysis of B19V, respectively (Supplementary Table S1).

### Genotype identification and phylogenetic analysis

Based on NS1-VP1u and the genome dataset of B19V, phylogenetic trees were constructed by the maximum likelihood (ML) approach in MEGA software. The phylogenetic tree was tested with 1000 bootstrap replications, and bootstrap values greater than 80% are indicated on the trees. The P-distances within and between genotypes/subgenotypes at the nucleotide level and the amino acid mutation rates in six proteins were calculated by using MEGA software, and nucleotide similarities were calculated by using BioEdit software (Version 7.0.5.1).

### Evolutionary rate and selection analysis

The genome dataset was analyzed with the BEAST package (version 1.10.4) to estimate the evolutionary rate of B19V and the BEAST tree of B19V was also conducted in this study. Initially, the nucleotide substitution model was determined by jModelTest2 (version 2.1.6) online software. With Bayesian skyline as coalescent tree priors, three different molecular clock models were implemented in the BEAST analysis, including strict clock, uncorrelated exponential relaxed clock, and uncorrelated log-normal relaxed clock. Log marginal likelihoods were determined by stepping-stone sampling (SS). The best fit models for the genome and six proteins of B19V were determined by Bayesian factor analysis. The Markov Chain Monte Carlo method was performed for 50 million generations and sampled so that 1,000 trees were generated. Finally, the convergence of the chains and the effective sample size (> 200) were determined by Tracer software (version 1.7.1), and the uncertainty of the parameter estimates was assessed by a 95% HPD interval.

Selection pressure was also analyzed in six proteins of B19V based on the genome dataset. DnaSP6 software (Version 6.0) was used to calculate the ω values (*ω* = *dN/dS*) of B19V, where *dN* represents the nonsynonymous substitution rate and *dS* represents the synonymous substitution rate. Gene-specific and site-specific selection pressures were measured as the value *dN/dS* at each codon site and estimated using four different codon-based maximum-likelihood methods (FEL, SLAC, FUBAR and MEME), and Tamura-Nei model (TrN) or Hasegawa-Kishino-Yano model (HKY85) were used as nucleotide substitution models^50^. All methods were obtained available at the DataMonkey online version of the Hyphy package (www.datamonkey.org) with significance levels set at *p* < 0.05, and the posterior probability of the FUBAR algorithm was > 0.95.

### Accession number

Seven genome sequences and three NS1-VP1u sequences of B19V in this study were submitted to GenBank with accession numbers OR533486 ~ OR533495 (Supplementary Table S1).

### Ethics approval and consent to participate

This study was approved by the ethical review committee of the National Institute for Viral Disease Control and Prevention, Chinese Centers for Disease Control and Prevention. The informed consents were signed by patients or their legal guardians.

### Supplementary Information

Supplementary Information.

## Data Availability

The data that support the findings of this study are available on request from the corresponding author. The data are not publicly available due to privacy or ethical restrictions.
