# Supplementary material for: The epidemiological and genetic characteristics of human parvovirus B19 in patients with febrile rash illnesses in China
Source: Sci Rep. 2023 Sep 23;13:15913. doi: 10.1038/s41598-023-43158-y (PMC10517975; doi:10.1038/s41598-023-43158-y)
Supplement: Supplementary file 1 — Supplementary Information. [file 41598_2023_43158_MOESM1_ESM.docx]

**Supplementary**

**Table S1.** The information of the sequences in B19V

| Accession Number | Strain name | Source/ Clinical manifestations | Country | Year | Genotype | Database | Reference ^a^ |
| --- | --- | --- | --- | --- | --- | --- | --- |
| AB030673.1 | N8 | aplastic crisis | Japan | 1986 | 1a | NS1-VP1u | ^1^ |
| AB030693.1 | Mi | rheumatoid arthritis | Japan | 1992 | 1a | NS1-VP1u |  |
| AB030694.1 | Rm | rheumatoid arthritis | Japan | 1992 | 1a | NS1-VP1u |  |
| AB126268.1 | AN56 | an infant's liver with congenital biliary atresia | Japan | 1998 | 1a | NS1-VP1u | ^2^ |
| AB126269.1 | AN66 | an infant's liver with fulminant hepatitis | Japan | 1997 | 1a | NS1-VP1u |  |
| AB126270.1 | AN85 | an infant's liver with fulminant hepatitis | Japan | 2000 | 1a | NS1-VP1u |  |
| AM490420.1 | B19Vs_LUX_35.01 | febrile rash illnesses | Luxembourg | 2001 | 1a | NS1-VP1u | ^3^ |
| AM490421.1 | B19Vs_LUX_31.02 | febrile rash illnesses | Luxembourg | 2002 | 1a | NS1-VP1u |  |
| AM490423.1 | B19Vs_LUX_19.04 | febrile rash illnesses | Luxembourg | 2004 | 1a | NS1-VP1u |  |
| JN211126.1 | 35628 | healthy donor blood | Netherlands | 2009 | 1a | NS1-VP1u | ^4^ |
| JN211155.1 | 140645 | healthy donor blood | Netherlands | 2003 | 1a | NS1-VP1u |  |
| KC013335.1 | 08BRSP3007 | leukaemia | Brazil | 2008 | 1a | NS1-VP1u | ^5^ |
| KR819781.1 | A108 | healthy donor plasma | China | 2013 | 1a | NS1-VP1u | ^6^ |
| KR819823.1 | A35 | healthy donor plasma | China | 2009 | 1a | NS1-VP1u |  |
| KR819836.1 | A48 | healthy donor plasma | China | 2009 | 1a | NS1-VP1u |  |
| KR819879.1 | C56 | healthy donor plasma | China | 2008 | 1a | NS1-VP1u |  |
| KT389469.1 | HZ92 | HIV positive patient | Zhejiang.China | 2015 | 1a | NS1-VP1u | ^7^ |
| KT389470.1 | HZ128 | HIV positive patient | Zhejiang.China | 2015 | 1a | NS1-VP1u |  |
| MN817182.1 | 43918 | febrile rash illnesses | Belarus | 2013 | 1a | NS1-VP1u | ^8^ |
| MH151119.1 | FX12 | healthy donor plasma | China | 2017 | 1a | NS1-VP1u | NA |
| MZ695231.1 | B19_664 | [kidney transplant](https://www.sciencedirect.com/topics/medicine-and-dentistry/kidney-graft) recipient | China | 2021 | 1a | NS1-VP1u | NA |
| MZ695232.1 | B19_681 | kidney transplant recipient | China | 2021 | 1a | NS1-VP1u |  |
| MH151120.1 | FX59 | healthy donor plasma | China | 2017 | 1b | NS1-VP1u | NA |
| DQ357064.1 | Vn147 | hepatitis B virus infection | VietNam | 2000-2002 | 1b | NS1-VP1u | ^9^ |
| DQ357065.1 | Vn115 | hepatitis B virus infection | VietNam | 2000-2002 | 1b | NS1-VP1u |  |
| KR819778.1 | A111 | healthy donor plasma | China | 2013 | 1b | NS1-VP1u | ^6^ |
| KR819832.1 | A44 | healthy donor plasma | China | 2009 | 1b | NS1-VP1u |  |
| AY044266.2 | LaLi | skin samples | Finland | - | 2 | NS1-VP1u | ^10^ |
| AY903437.2 | IM_81 | healthy donor blood | Germany | - | 2 | NS1-VP1u | ^11^ |
| HQ340602.1 | B19 | myocarditis and cardiomyopathy | Germany | 2009 | 2 | NS1-VP1u | ^12^ |
| FN295740.1 | BFA_51235 | febrile rash illnesses | Burkina_Faso | 2007-2008 | 3a | NS1-VP1u | ^3^ |
| FN295717.1 | Osh06_112 | febrile rash illnesses | Kyrgyzstan | 2006 | 3b | NS1-VP1u |  |
| KR819847.1 | A59 | healthy donor plasma | China | 2009 | 3b | NS1-VP1u | ^6^ |
| KR819864.1 | B13 | healthy donor plasma | China | 2009 | 3b | NS1-VP1u |  |
| AY386330.1 | J35 | sickle cell anemia | USA | 2003 | 1a | genome | ^13^ |
| DQ293995.2 | C39 | healthy donor blood | Belgium | 2000 | 1a | genome | ^14^ |
| KM393163.1 | KU2 | healthy donor plasma | USA | 2013 | 1a | genome | ^15^ |
| KM393164.1 | KU3 | healthy donor plasma | USA | 2013 | 1a | genome |  |
| KM393165.1 | KU4 | healthy donor plasma | USA | 2013 | 1a | genome |  |
| KM393166.1 | KU5 | healthy donor plasma | USA | 2013 | 1a | genome |  |
| KM393167.1 | KU8 | healthy donor plasma | USA | 2013 | 1a | genome |  |
| KM393168.1 | KU11 | healthy donor plasma | USA | 2013 | 1a | genome |  |
| KM393169.1 | KU12 | healthy donor plasma | USA | 2013 | 1a | genome |  |
| KR005644.1 | MJ21_RS8 | migratory arthritis | Serbia | 2012 | 1a | genome | ^16^ |
| KT310174.1 | HZ1 | the M. pneumoniae | Zhejiang.China | 2014 | 1a | genome | ^7^ |
| M13178.1 | Au | aplastic crisis | USA | 1982 | 1a | genome | ^17,18^ |
| M24682.1 | Wi | healthy donor blood | England | 1973 | 1a | genome | ^17,19^ |
| MH201455.1 | BX1 | healthy donor blood | USA | 2009 | 1a | genome | ^20^ |
| MH201456.1 | BX2 | healthy donor blood | USA | 2009 | 1a | genome |  |
| MK989716.1 | 20692_3_B19 | severe pneumonia and anaemia | Kenya | 2015 | 1a | genome | ^21^ |
| MN765167.1 | SAfia_387D | acute febrile illness | Tanzania | 2015 | 1a | genome | ^22^ |
| MN765168.1 | SAfia_026D | acute febrile illness | Tanzania | 2015 | 1a | genome |  |
| MN765169.1 | SAfia_676D | acute febrile illness | Tanzania | 2015 | 1a | genome |  |
| MN765170.1 | SAfia_386D | acute febrile illness | Tanzania | 2015 | 1a | genome |  |
| MT410187.1 | AA_04 | emoral bone of recently deceased individuals | Finland | 2015 | 1a | genome | ^23^ |
| Z68146.1 | Stu | blood donors | England | 1993 | 1a | genome | ^24^ |
| Z70560.1 | I_1 | acute infection of B19V | Germany | 1994 | 1a | genome | ^25^ |
| FJ591158.1 | KU1 | healthy donor plasma | USA | 2008 | 1a | genome | NA |
| MZ695225.1 | B19_490 | kidney transplant recipient | Shanghai.China | 2021 | 1a | genome | NA |
| MZ695227.1 | B19_564 | [kidney transplant](https://www.sciencedirect.com/topics/medicine-and-dentistry/kidney-graft) recipient | Shanghai.China | 2021 | 1a | genome |  |
| MZ695229.1 | B19_582 | kidney transplant recipient | Shanghai.China | 2021 | 1a | genome |  |
| MH151117.1 | 1b | healthy donor blood | China | 2017 | 1b | genome | NA |
| AY064475.1 | A6_c2 | anemic HIV | Italy | 1991 | 2 | genome | ^26^ |
| AY064476.1 | A6_c8 | anemic HIV | Italy | 1991 | 2 | genome |  |
| DQ333426.1 | BN31.2 | liver specimens | Germany | - | 2 | genome | ^27^ |
| DQ333427.1 | BN32.2 | liver specimens | Germany | - | 2 | genome |  |
| MT410184.1 | AA_01 | emoral bone of recently deceased individuals | Finland | 2015 | 2 | genome | ^23^ |
| AJ249437.1 | V9 | aplastic anemia | France | 1995 | 3a | genome | ^28^ |
| AY582125.2 | Gh3051 | healthy donor blood | Ghana | 2002 | 3a | genome | ^14^ |
| DQ234769.2 | R0416 | severe anemia | Ghana | 2004 | 3a | genome |  |
| DQ234771.2 | R0227 | severe anemia | Ghana | 2003 | 3a | genome |  |
| DQ234775.2 | D1599 | severe anemia | Ghana | 2005 | 3a | genome |  |
| FJ265736.1 | P1 | healthy donor plasma | USA | 2007 | 3a | genome | ^29^ |
| AY083234.1 | D91.1 | aplastic crisis, G6PD deficit, minor thalassemia | France | 1991 | 3b | genome | ^30^ |
| AY582124.2 | Gh2768 | healthy donor blood | Ghana | 2002 | 3b | genome | ^14^ |
| DQ234778.2 | R0693 | severe anemia | Ghana | 2004 | 3b | genome |  |
| DQ234779.2 | R0748 | severe anemia | Ghana | 2004 | 3b | genome |  |
| DQ408302.1 | BN58.3 | liver specimens | Germany | - | 3b | genome | ^27^ |
| DQ408303.1 | BN59.3 | liver specimens | Germany | - | 3b | genome |  |
| DQ408304.1 | BN60.3 | liver specimens | Germany | - | 3b | genome |  |
| DQ408305.1 | BN30.3 | liver specimens | Germany | - | 3b | genome |  |
| MN765171.1 | SAfia_743D | acute febrile illness | Tanzania | 2015 | 3b | genome | ^22^ |
| OK482570.1 | BDN_17_344 | meningoencephalitis | India | 2017 | 3b | genome | ^31^ |
| OK482571.1 | BDN_17_357 | meningoencephalitis | India | 2017 | 3b | genome |  |
| OK482578.1 | BDN_18_200 | meningoencephalitis | India | 2018 | 3b | genome |  |
| OR533486 | Henan-113 | febrile rash illnesses | Henan.China | 2018 | 1a | genome | In study |
| OR533487 | Shaanxi-236 | febrile rash illnesses | Shaanxi.China | 2019 | 1a | genome |  |
| OR533488 | Shaanxi-77 | febrile rash illnesses | Shaanxi.China | 2019 | 1a | genome |  |
| OR533489 | Shanghai-358 | febrile rash illnesses | Shanghai.China | 2019 | 1a | genome |  |
| OR533490 | Shanghai-52 | febrile rash illnesses | Shanghai.China | 2018 | 1a | genome |  |
| OR533491 | Shanxi-46 | febrile rash illnesses | Shanxi.China | 2011 | 1a | genome |  |
| OR533492 | Shanxi-T1 | febrile rash illnesses | Shanxi.China | 2013 | 1a | genome |  |
| OR533493 | Shanghai-87 | febrile rash illnesses | Shanghai.China | 2018 | 1a | NS1-VP1u |  |
| OR533494 | Shanxi-11 | febrile rash illnesses | Shanxi.China | 2009 | 1a | NS1-VP1u |  |
| OR533495 | Shanxi-9 | febrile rash illnesses | Shanxi.China | 2009 | 1a | NS1-VP1u |  |

a: Accession number of each sequence record in nucleotide database of NCBI; information on strain name, source/ clinical manifestations, country, and year are contained within linked references; NA: not available, the sequence was downloaded from NCBI; -: Temporal information is unclear not included in the evolutionary rate and selection pressure analysis.

**Table S2.** The genotype-specific amino acid sites among three genotypes of B19V in six proteins

| Genotype | NS1 (1-671AA) | | | | | | | | | | | | | | | | | | | | | | | | | | | | |
| --- | --- | --- | --- | --- | --- | --- | --- | --- | --- | --- | --- | --- | --- | --- | --- | --- | --- | --- | --- | --- | --- | --- | --- | --- | --- | --- | --- | --- | --- |
|  | 10 | 26 | 40 | 93 | 113 | 168 | 178 | 184 | 185 | 186 | 187 | 189 | 192 | 197 | 198 | 201 | | 213 | 214 | 277 | 285 | 344 | 473 | 501 | 505 | 536 | | 603 | |
| 1 | **V** | **L** | **T** | **P** | T | **T** | K | **A/G** | **I** | **N** | **D** | S/C | A/P | **G/S** | **T** | **E** | | **S** | **I/M** | L | **M** | **S** | **D** | T/A | **T** | **T** | | Q | |
| 2 | I | M | S | A | **N** | S | K | N | T | D | **T** | **N** | A/G | C | G | D | | G | L | **M** | T | T | N | **V** | A/P | S | | Q | |
| 3 | I | M | S | A | T | S | **R** | N | A/T | D | **N/S/H** | T/S/A | **T** | C | G/A | D | | G | L | L | T | T | S/N | T | P/S | S | | **E** | |
|  |  |  |  |  |  |  |  |  |  |  |  |  |  |  |  |  | |  |  |  |  |  |  |  |  |  | |  | |
| Genotype | VP1 (1-781AA) | | | | | | | | | |  | VP2 (1-554AA) | | | | |  |  |  |  |  |  |  |  |  | |  | |  |
|  | 4 | 5 | 17 | 21 | 68 | 72 | 248 | 418 | 521 | 616 |  | 21 | 191 | 294 | 389 |  | |  |  |  |  |  |  |  |  |  | |  | |
| 1 | E/K/N/Q | S | K | **Q** | **N** | S | V/A | D | V | M |  | V/A | D | V | M |  | |  |  |  |  |  |  |  |  |  | |  | |
| 2 | E/K | S/A | K | K | S | **D** | V/A | D | V | **I** |  | V/A | D | V | **I** |  | |  |  |  |  |  |  |  |  |  | |  | |
| 3 | **T/A** | **T** | **Q** | K | S | N/S | **T** | **E** | **I** | M |  | **T** | **E** | **I** | M |  | |  |  |  |  |  |  |  |  |  | |  | |
|  |  |  |  |  |  |  |  |  |  |  |  |  |  |  |  |  | |  |  |  |  |  |  |  |  |  | |  | |
| Genotype | 7.5KDa (1-74AA) | | | | | | |  | X (1-81AA) | |  | 11KDa (1-94AA) | | | | | | | | | | | | | | |  | |  |
|  | 12 | 13 | 37 | 41 | 47 | 54 | 74 |  | 17 | 58 |  | 18 | 37 | 40 | 50 | 56 | | 58 | 60 | 61 | 64 | 66 | 69 | 71 | 90 |  | |  | |
| 1 | **P** | **Q** | **L** | **P** | **L** | R | **L** |  | M | P |  | **Q** | **C** | **K** | **L** | **I** | | Q | **N** | **T** | **I** | **N/Y** | **F** | **Y** | **Q** |  | |  | |
| 2 | S | P | S | L | V | **H** | W |  | I/M | P |  | L | T | N | V | R | | **E** | **C/L** | V/A | T | A | Y | L | T |  | |  | |
| 3 | P | P | S | L | V | R | W |  | **V/L** | **L** |  | L | T | N/H | V | S/R | | Q | **S/F** | V/I | T | A | Y | L | T |  | |  | |

Bold black represents the characteristic variation of the site.


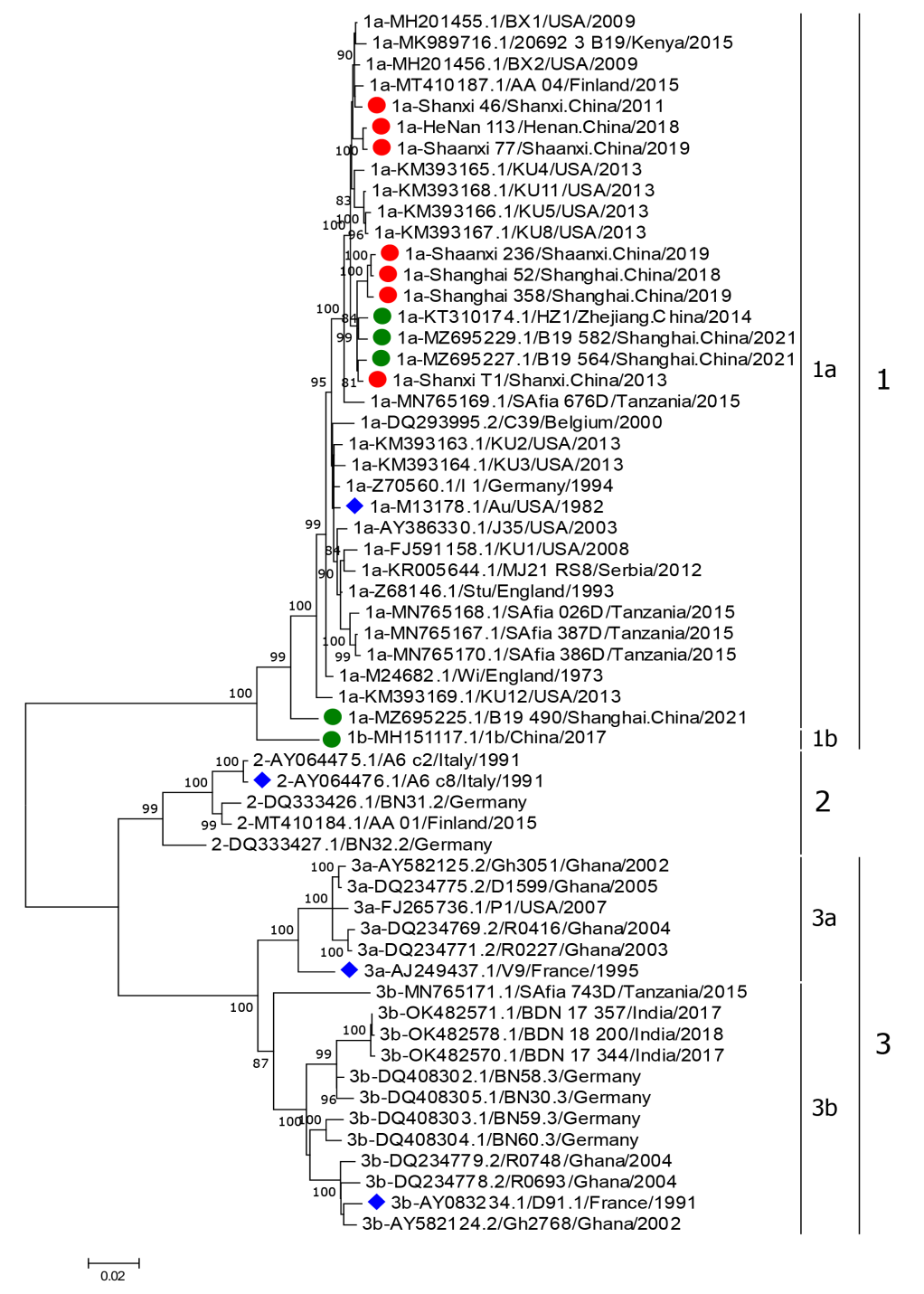


**Figure S1.** Phylogenetic tree of maximum likelihood (ML) method based on the B19V genome sequences.

A phylogenetic tree was constructed with the maximum likelihood method. Bootstrap values above 80 % are shown (1000 replicates). The blue diamond represents the reference sequences of genotype or subgenotype; the green solid circle represents the Chinese representative sequences; the red solid circle represents the sequence obtained in this study.

**
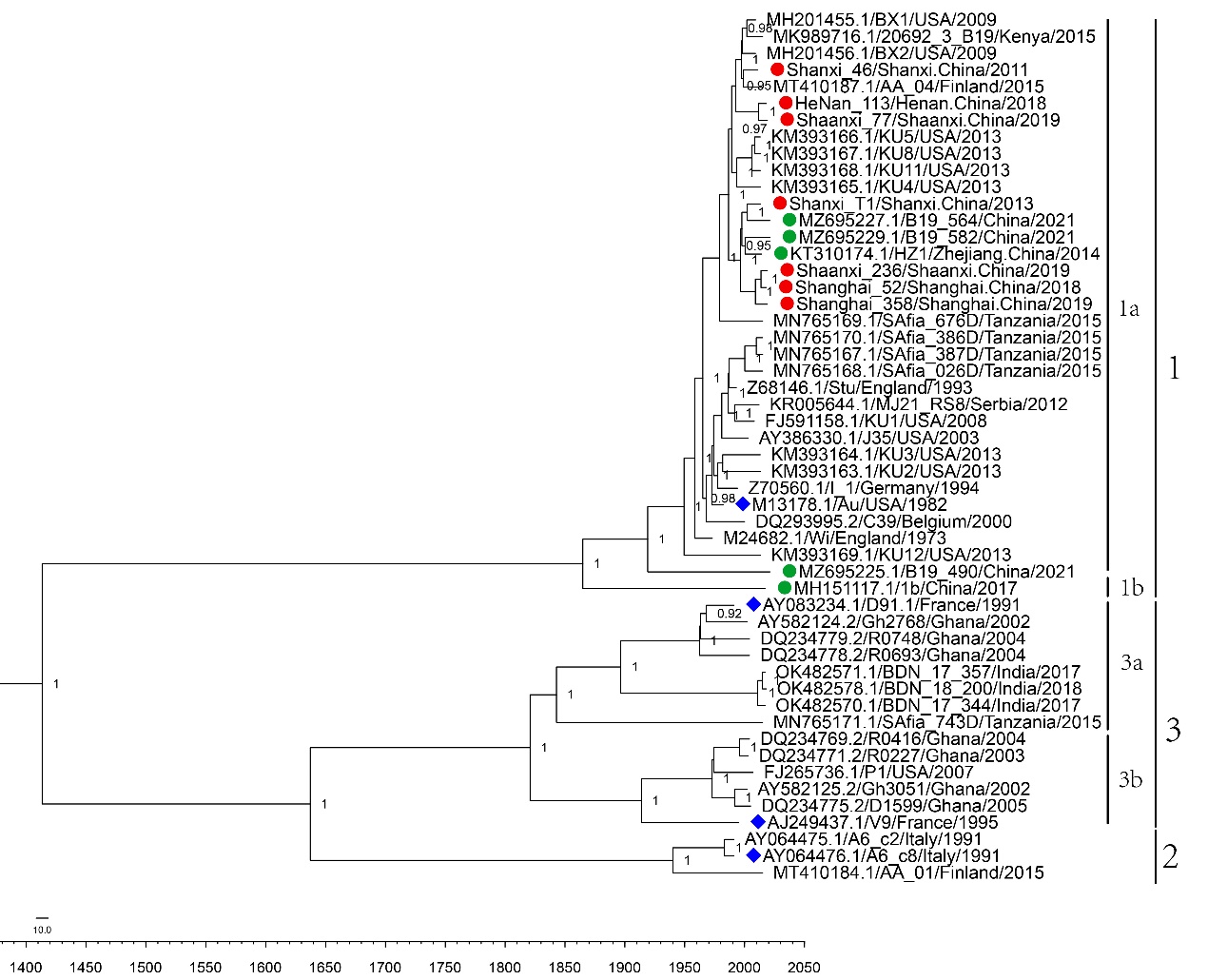
**

**Figure S2.** Beast tree based on the B19V genome sequences.

BEAST tree based on 52 B19V genome sequences. The blue diamond represents the reference sequences of genotype or subgenotype; the green solid circle represents the Chinese representative sequences; the red solid circle represents the sequence obtained in this study.

**Table S3.** List of clinical specimen collection information of FRI cases in China

| Region | Province | Sampling time (years) | Sample size (number) |
| --- | --- | --- | --- |
| Northern | Xinjiang Uygur Autonomous Region | 2013-2014 | 4 |
|  | Beijing | 2009-2010、2013-2015、2017-2020 | 498 |
|  | Hebei | 2009、2011-2014、2018-2020 | 433 |
|  | Henan | 2018-2019 | 6 |
|  | Shaanxi | 2010-2011、2013-2015、2018-2021 | 328 |
|  | Shandong | 2013-2015、2018-2020 | 548 |
|  | Shanxi | 2009-2011、2013-2015、2017-2019 | 867 |
| Southern | Anhui | 2009-2010、2017-2021 | 694 |
|  | Hunan | 2010、2020 | 14 |
|  | Shanghai | 2010、2018-2019 | 428 |
| Total |  | 2009-2021 | 3820 |

**Table S4.** The list of nested PCR amplification primers for B19V genome

| Segments | Primers | Sequence (5'-3') | Length (bp) | Position ^a^ |
| --- | --- | --- | --- | --- |
| 1 | F1w | GCATCTGATTTGGTGTCTTC | 475 | 353-372 |
|  | F1n | TTCCCGCCTTATGCAAATGG |  | 395-414 |
|  | R1n | ACCACATGAATATGATAGCC |  | 850-869 |
|  | R1w | CACTGTTAGGTTTCTGGGGT |  | 890-909 |
| 2 | F2w | CTTCTAATGTTCTGGACTGTGC | 712 | 647-668 |
|  | F2n | GGACTGTGCTAACGATAACTGG |  | 660-681 |
|  | R2n | ACTTCCACTGTGACTACTGC |  | 1352-1371 |
|  | R2w | CATAACCTGCTCAAAGTCTG |  | 1451-1470 |
| 3 | F3w | CAGAGGTTGTGCCATTTA | 920 | 1214-1231 |
|  | F3n | TAATGGGAAGGGAACTAAGG |  | 1239-1249 |
|  | R3n | CACCACCACTGCTGCTGATA |  | 2139-2158 |
|  | R3w | GCGGGGTTTCAGTGTTCCA |  | 2215-2233 |
| 4^*^ | F4w | TGGCTAACTTGGTGTAATGC | 1,166 | 2002-2021 |
|  | F4n | ACTGGGCAATAAACTACACT |  | 2045-2064 |
|  | R4n | ACTACTTGTGCTTGAAACCC |  | 3182-3210 |
|  | R4w | TTCTGAGGCGTTGTAAGCGG |  | 3273-3292 |
| 5 | F5w | CAGTCATGCAGAACCTAGAG | 837 | 2917-2936 |
|  | F5n | CCTGGGCAAGTTAGCGTA |  | 2975-2992 |
|  | R5n | AGTATCCTGACCTTGCCCTA |  | 3792-3811 |
|  | R5w | GTTCTGGGGCTAAAGTATCC |  | 3805-3824 |
| 6 | F6w | TGGAAGTATAGCTCCTGATG | 773 | 3637-3656 |
|  | F6n | AGCTCCTGATGCTTTAACTG |  | 3646-3665 |
|  | R6n | CCACTCCTTGCTGATACTC |  | 4400-4418 |
|  | R6w | TCTACCCACTCCTTGCTGA |  | 4405-4423 |
| 7 | F7w | TACAAAGGAGGGAGACAGC | 614 | 4198-4216 |
|  | F7n | GCCAGTGTCTCAGCCATACC |  | 4297-4316 |
|  | R7n | GTCTGTGGTGTTGTTTTGC |  | 4892-4910 |
|  | R7w | GTTTAGTTACGCATCCTGG |  | 4992-5010 |
| 8 | F8w | TTAGTTCAGTATGCCGTGG | 575 | 4736-4754 |
|  | F8n | CTACGGGACGGTGGAATCCT |  | 4800-4819 |
|  | R8n | GCCCAGAGCCAACCCTAATT |  | 5355-5374 |
|  | R8w | CTTAGTGGCACGTCAACCC |  | 5384-5402 |

a: Position numbered according to the B19V reference sequence NC_000883.2; * the pair of the primers for genotype identification; F, forward primer; R, reverse primer; w, the first-round primer; n, the second-round primer.

**Reference**

1. Takasawa N,Munakata Y,Ishii KK,Takahashi Y,Takahashi M,Fu Y, et al. Human parvovirus B19 transgenic mice become susceptible to polyarthritis, *J Immunol,* 2004; 173:4675-4683. doi:10.4049/jimmunol.173.7.4675.

2. Abe K,Kiuchi T,Tanaka K,Edamoto Y,Aiba N,Sata T. Characterization of erythrovirus B19 genomes isolated in liver tissues from patients with fulminant hepatitis and biliary atresia who underwent liver transplantation, *Int J Med Sci,* 2007; 4:105-109. doi:10.7150/ijms.4.105.

3. Hubschen JM,Mihneva Z,Mentis AF,Schneider F,Aboudy Y,Grossman Z, et al. Phylogenetic analysis of human parvovirus b19 sequences from eleven different countries confirms the predominance of genotype 1 and suggests the spread of genotype 3b, *J Clin Microbiol,* 2009; 47:3735-3738. doi:10.1128/JCM.01201-09.

4. Molenaar-de Backer MW,Lukashov VV,van Binnendijk RS,Boot HJ,Zaaijer HL. Global co-existence of two evolutionary lineages of parvovirus B19 1a, different in genome-wide synonymous positions, *PLoS One,* 2012; 7:e43206. doi:10.1371/journal.pone.0043206.

5. da Costa AC,Bendit I,de Oliveira ACS,Kallas EG,Sabino EC,Sanabani SS. Investigation of human parvovirus B19 occurrence and genetic variability in different leukaemia entities, *Clin Microbiol Infect,* 2013; 19:E31-E43. doi:10.1111/1469-0691.12058.

6. Jia J,Ma Y,Zhao X,Huangfu C,Zhong Y,Fang C, et al. Existence of various human parvovirus B19 genotypes in Chinese plasma pools: genotype 1, genotype 3, putative intergenotypic recombinant variants and new genotypes, *Virol J,* 2016; 13:155. doi:10.1186/s12985-016-0611-6.

7. Zhang L,Cai C,Pan F,Hong L,Luo X,Hu S, et al. Epidemiologic study of human parvovirus B19 infection in East China, *J Med Virol,* 2016; 88:1113-1119. doi:10.1002/jmv.24459.

8. Yermalovich MA,Dronina AM,Semeiko GV,Samoilovich EO,Khrustalev VV,Sausy A, et al. Comprehensive surveillance data suggest a prominent role of parvovirus B19 infection in Belarus and the presence of a third subtype within subgenotype 1a, *Sci Rep,* 2021; 11:1225. doi:10.1038/s41598-020-79587-2.

9. Toan NL,Duechting A,Kremsner PG,Song LH,Ebinger M,Aberle S, et al. Phylogenetic analysis of human parvovirus B19, indicating two subgroups of genotype 1 in Vietnamese patients, *J Gen Virol,* 2006; 87:2941-2949. doi:10.1099/vir.0.82037-0.

10. Hokynar K,Soderlund-Venermo M,Pesonen M,Ranki A,Kiviluoto O,Partio EK, et al. A new parvovirus genotype persistent in human skin, *Virology,* 2002; 302:224-228. doi:10.1006/viro.2002.1673.

11. Blumel J,Eis-Hubinger AM,Stuhler A,Bonsch C,Gessner M,Lower J. Characterization of Parvovirus B19 genotype 2 in KU812Ep6 cells, *J Virol,* 2005; 79:14197-14206. doi:10.1128/JVI.79.22.14197-14206.2005.

12. Bock CT,Duchting A,Utta F,Brunner E,Sy BT,Klingel K, et al. Molecular phenotypes of human parvovirus B19 in patients with myocarditis, *World J Cardiol,* 2014; 6:183-195. doi:10.4330/wjc.v6.i4.183.

13. Zhi N,Zadori Z,Brown KE,Tijssen P. Construction and sequencing of an infectious clone of the human parvovirus B19, *Virology,* 2004; 318:142-152. doi:10.1016/j.virol.2003.09.011.

14. Parsyan A,Kerr S,Owusu-Ofori S,Elliott G,Allain JP. Reactivity of genotype-specific recombinant proteins of human erythrovirus B19 with plasmas from areas where genotype 1 or 3 is endemic, *J Clin Microbiol,* 2006; 44:1367-1375. doi:10.1128/JCM.44.4.1367-1375.2006.

15. Qiu Y,Zhao Z,Qiu J. Sequences of Seven Complete Genomes of Human Parvovirus B19, *Microbiol Resour Announc,* 2018; 7:doi:10.1128/MRA.00885-18.

16. Stamenkovic GG,Cirkovic VS,Siljic MM,Blagojevic JV,Knezevic AM,Joksic ID, et al. Substitution rate and natural selection in parvovirus B19, *Sci Rep,* 2016; 6:35759. doi:10.1038/srep35759.

17. Cotmore SF,Tattersall P. Characterization and molecular cloning of a human parvovirus genome, *Science,* 1984; 226:1161-1165. doi:10.1126/science.6095448.

18. Shade RO,Blundell MC,Cotmore SF,Tattersall P,Astell CR. Nucleotide sequence and genome organization of human parvovirus B19 isolated from the serum of a child during aplastic crisis, *J Virol,* 1986; 58:921-936. doi:10.1128/JVI.58.3.921-936.1986.

19. Blundell MC,Beard C,Astell CR. In vitro identification of a B19 parvovirus promoter, *Virology,* 1987; 157:534-538. doi:10.1016/0042-6822(87)90296-0.

20. Matz B,Kupfer B,Kallies R,Kulshammer M,Flotenmeyer M,Kreil TR, et al. Secondary structure of DNA released from purified capsids of human parvovirus B19 under moderate denaturing conditions, *J Gen Virol,* 2019; 100:812-827. doi:10.1099/jgv.0.001253.

21. Phan MVT,Agoti CN,Munywoki PK,Otieno GP,Ngama M,Kellam P, et al. Identification of missed viruses by metagenomic sequencing of clinical respiratory samples from Kenya, *Sci Rep,* 2022; 12:202. doi:10.1038/s41598-021-03987-1.

22. Cordey S,Laubscher F,Hartley MA,Junier T,Keitel K,Docquier M, et al. Blood virosphere in febrile Tanzanian children, *Emerg Microbes Infect,* 2021; 10:982-993. doi:10.1080/22221751.2021.1925161.

23. Toppinen M,Pratas D,Vaisanen E,Soderlund-Venermo M,Hedman K,Perdomo MF, et al. The landscape of persistent human DNA viruses in femoral bone, *Forensic Sci Int Genet,* 2020; 48:102353. doi:10.1016/j.fsigen.2020.102353.

24. Hicks KE,Cubel RC,Cohen BJ,Clewley JP. Sequence analysis of a parvovirus B19 isolate and baculovirus expression of the non-structural protein, *Arch Virol,* 1996; 141:1319-1327. doi:10.1007/BF01718833.

25. Hemauer A,von Poblotzki A,Gigler A,Cassinotti P,Siegl G,Wolf H, et al. Sequence variability among different parvovirus B19 isolates, *J Gen Virol,* 1996; 77 ( Pt 8):1781-1785. doi:10.1099/0022-1317-77-8-1781.

26. Nguyen QT,Wong S,Heegaard ED,Brown KE. Identification and characterization of a second novel human erythrovirus variant, A6, *Virology,* 2002; 301:374-380. doi:10.1006/viro.2002.1585.

27. Schneider B,Hone A,Tolba RH,Fischer HP,Blumel J,Eis-Hubinger AM. Simultaneous persistence of multiple genome variants of human parvovirus B19, *J Gen Virol,* 2008; 89:164-176. doi:10.1099/vir.0.83053-0.

28. Nguyen QT,Sifer C,Schneider V,Allaume X,Servant A,Bernaudin F, et al. Novel human erythrovirus associated with transient aplastic anemia, *J Clin Microbiol,* 1999; 37:2483-2487. doi:10.1128/JCM.37.8.2483-2487.1999.

29. Rinckel LA,Buno BR,Gierman TM,Lee DC. Discovery and analysis of a novel parvovirus B19 Genotype 3 isolate in the United States, *Transfusion,* 2009; 49:1488-1492. doi:10.1111/j.1537-2995.2009.02160.x.

30. Servant A,Laperche S,Lallemand F,Marinho V,De Saint Maur G,Meritet JF, et al. Genetic diversity within human erythroviruses: identification of three genotypes, *J Virol,* 2002; 76:9124-9134. doi:10.1128/jvi.76.18.9124-9134.2002.

31. Pattabiraman C,Prasad P,Sudarshan S,George AK,Sreenivas D,Rasheed R, et al. Identification and Genomic Characterization of Parvovirus B19V Genotype 3 Viruses from Cases of Meningoencephalitis in West Bengal, India, *Microbiol Spectr,* 2022; 10:e0225121. doi:10.1128/spectrum.02251-21.
